# Comparison of prognostic indices in symptomatic and screen-detected invasive breast cancer in Asian and Caucasian women

**DOI:** 10.1186/bcr3800

**Published:** 2015-11-05

**Authors:** Andrew Steele, Anil Jain, Navneet Randhawa, Philip Foden, Julie Morris

**Affiliations:** 1 The University of Manchester, UK; 2 The Nightingale Centre and Genesis Prevention Centre, Manchester, UK

## Introduction

In the UK, ethnic minority groups have reported lower awareness of breast screening and have presented with breast cancer symptoms later than Caucasian women. Our study compared prognostic indices in symptomatic and screen-detected breast cancer between Asian and Caucasian patients.

## Methods

Of the 310 Asian women diagnosed with breast cancer between 1999 and 2014 in the Asian Breast Cancer Database, 217 with invasive cancer were selected (57 screen-detected and 160 symptomatic). Data on invasive tumour size, grade, lymph node status and NPI were compared with age and mode-matched Caucasian breast cancer patients.

## Results

Asian symptomatic women had larger invasive tumours (median 25.0 mm, IQR 17.1−35.8 mm), compared with Caucasian patients (median 17.0 mm, IQR 12.0−26.4 mm) (*p* <0.001); higher proportions of grade 3 tumours (64.4 %) (*p *= 0.007) and with more than one lymph node involved (46.2 %) (*p *= 0.004), compared with Caucasian patients (48.8 % and 30.0 % respectively); worse NPI scores (median 4.6, IQR 4.3−5.6), compared with Caucasian patients (median 4.3, IQR 3.3−4.7) (*p* <0.001); and higher proportions with poor prognosis (33.8 %), compared with Caucasian patients (11.9 %) (*p* <0.001). Multivariable analysis showed invasive grade and tumour size were statistically significant independent discriminators with lymph node status as borderline significant. However, there was no statistically significant difference between the ethnic groups for screen-detected invasive tumours.

## Conclusion

Prognostic indices in Asian women were worse in symptomatic breast cancer, but similar in screen-detected invasive cancer, compared with age-matched Caucasian women. Greater initiatives need to be implemented to promote breast cancer awareness, education and screening among the Asian ethnic minorities.

